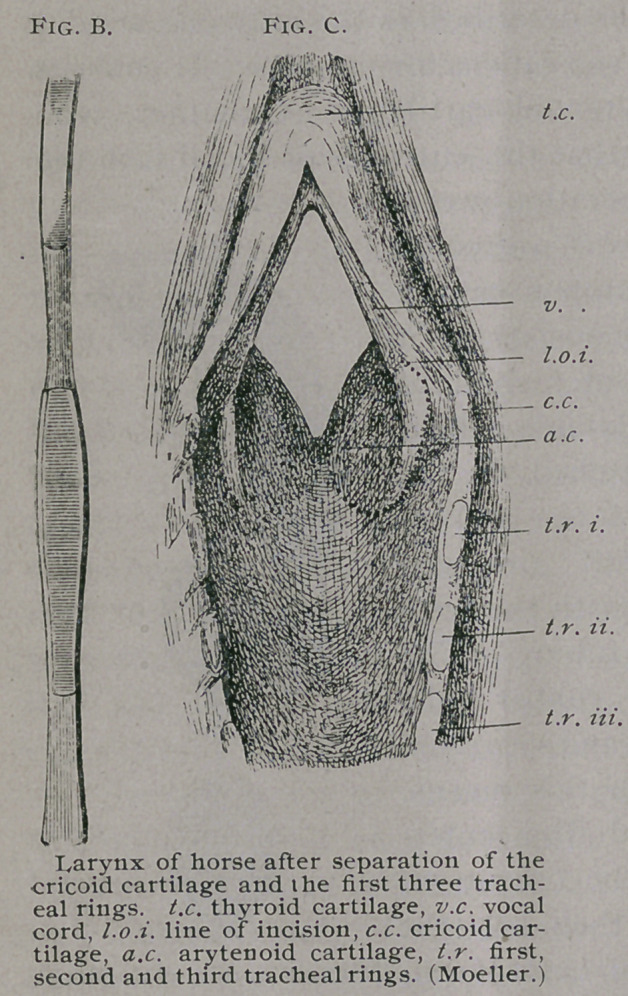# Roaring (Hemiplegia Laryngis)1This is the term given by H. Moeller in his work, Das Kehlkopfpfeifen der Pferde und seine operative Behandelung. Stuttgart, 1888. Fleming terms it Laryngismus Paralyticus.

**Published:** 1891-01

**Authors:** Leo Breisacher


### ROARING.

### (*Hemiplegia Laryngis.*)[1]

By Leo Breisacher, V.M.D.

Within the last year much has been written about the operation known as laryngotomy. The chaotic results obtained by various surgeons are bewildering, and to persons not acquainted with the facts of this *matter* the various claims as to the originator of this operation are misleading. Among veterinarians, as among professional men of different calling, one meets with a certain degree of strife and rivalry. Against honorable and *pure* rivalry no objection can be raised. When, however, overzealous individuals in their desire to emulate, use means which give them publicity and notoriety, to which they righteously have no claim, an interception becomes necessary. Leaving aside the plagiarism which has been practiced in connection with laryngotomy, I ask you, what have we gleaned from the various writings on the subject? and can but answer, nothing conducive of a proper and lucid conception of it. If laryngotomy is to be universally practiced, the profession should be presented with the RECORDS and results of the different operations, so that it can thoroughly and rationally test the efficiency of operative interferences as a means of relieving roaring. To-day, laryngotomy bids fair to fall into disrepute as it did twenty-six years ago, and only because its *modus operandi* has not been properly promulgated. The fact that eminent veterinarians fail with this operation leads us to suspect that the success of "lesser lights," in many cases, is rather due to a combination of accidental circumstances than to an actual comprehension of the subject. To accurately and efficiently perform laryngotomy considerable dexterity is necessary, which should be cultivated on the cadaver. Ere describing the operation proper, we would like to summarize in a short manner a few interesting facts which are linked to laryngotomy.

Galen and Riolan (1612) performed section of the recurrent

[1] This is the term given by H. Moeller in his work, Das Kehlkopfpfeifen der Pferde und seine operative Behandelung. Stuttgart, 1888. Fleming terms it Laryngismus Paralyticus.

laryngeal nerve on swine, so that their cries would not be heard while being "stuck." Lenglois, in 1812, through his experiments, first directed attention to the recurrent laryngeal nerve as a factor in the production of roaring. Godine (1811) is given the credit of having first intimated that roaring was due to an immobile arytenoid cartilage. Youatt (1833) was the first to describe the unilateral atrophy of the laryngeal muscles in roaring. Not only is the date of the first operation of laryngotomy but also its instigator often confounded or erroneously given. F. Guenther in 1823 experimentally produced roaring in horses. He busied himself with this subject until 1830. K. Guenther, a son of the above-named, was the originator of laryngotomy, performing the first operation in 1863. In 1885 Prof. Moeller rekindled an interest for laryngeal surgery by instituting a series of experiments relative to laryngotomy. He first tested the methods of Guenther, which he abandoned, although they proved beneficial in many instances; but again adopted them, with modifications, after having tested some newly-devised procedures. As will be seen later on, Moeller's labor has been crowned by the most brilliant results. To dwell upon the anatomy and physiology of the larynx would be superfluous, our object being only to rehearse this subject from a surgical standpoint. Of the various factors and conditions—such as enlarged lymphatic glands, polyps, calcification and contraction of the larynx, which occasion in some instances symptoms similar to those of laryngeal palsy—the reader is acquainted with. Here, where we are only dealing with hemiplegia laryngis—or laryngismus paralyticus, as you may choose to call it—only the principal measures for the recognition of this trouble will be dealt with. By applying the various tests it is almost impossible to err in a diagnosis. To locate the roaring in the larynx is far less difficult than to define the the seat of the trouble when the larynx has been found to be free from abnormalities. Turning the head of the animal to the side opposite to that on which the atrophy of the laryngeal muscles exists, increases the respiratory sound. The noise (roaring) is generally an inspiratory one, but at times also expiratory. (In tracheal and laryngeal tumors the expiratory sound is louder than the inspiratory one.) Pressure on the palsied side of the larynx increases the roaring. By this method Moeller diagnosed, by exclusion, fibrous thickening of the larynx. The experiments of Guenther seem to prove that extension of the guttural pouches cannot occasion abnormal respiratory sounds likely to be confounded with roaring. Thickening of the nasal mucous membrane causes a sniffling sound. The trouble is usually unilateral, and can be diagnosed by closing one nostril and then the other. But we have already wandered from our laryngeal trouble, which we promised not to do.

As to the primary causes of roaring many variegated tenets exist, which we will only review hurriedly. We have, for instance, pulsation of the aorta ; displacement of the heart, which is by some authorities claimed to take place during growth of the animal. Both of the preceding conditions, we need hardly mention, are supposed to act upon the inferior laryngeal nerve. Again, stretching of the nerve-trunk during growth of the cervical structures is by some looked upon as a factor in the production of roaring. Some hold that the above supposition explains why roaring is oftener met with in the male, where, as is known, the cervical structures assume a greater length, than in female horse. Training, by increasing the bloodvessel calibre, is held to be the cause of roaring in race animals—the increased bloodvessel calibre causing tension of the fascia-endothoracica, a strand of fascia extending from the pericardium to the trachea and covering the inferior laryngeal nerve. As is seen, many conditions can interfere with or wholly extinguish the functions of the inferior laryngeal nerve, which is the motor nerve of the entire laryngeal muscles with the exception of the crico-thyroid muscle. Moeller mentions that the greatest number of cases of roaring occur or develop between the age of three and six years, which points to the possibility that an uncomplemental growth between the nerve-trunk and the remaining cervical structures is in some cases the cause of the trouble. But here speculation has such a vast space to parade in that many theories can with little difficulty be adjusted, one apparently as valid as the other. That roaring can be transmitted from generation to generation has long been recognized. However, to what extent one is justified in withholding a roarer from the breeding-harem is not as yet quite clear. Where roaring can positively be traced to a thoracic trouble, or some other accidental cause, we are of the opinion that no great fears need be entertained. Nevertheless, hopes of evolving a valuable strain of animals from a roarer should never be cherished. On the other hand, when roaring arises during or after a course of training or during growth of the animal, or where no accidental causes can be traced, the animal should be pronounced unfit for breeding purposes. Norfolk and Suffolk breeders were at one time assured that roaring was not hereditary, which had deplorable effects upon the breeding interests of those districts. It is evident that with our new method of relieving roarers, great care should be exercised in examining animals destined for the breeding ranks. Roaring develops equally slow, whether arising in young animals or as the result of pneumonia and kindred affections, so that no light can be shed on the matter from this source. Two cases have been recorded in which roaring developed itself with fabulous rapidity, both during violent exercise, one a saddle the other a draft animal. As already mentioned, K. Guenther, in 1863, was the first to attempt a surgical operation for the relief of roaring. He first attempted partial excision of both vocal cords. Deriving no benefit from this procedure he extirpated the entire vocal cord of the affected side of the larynx. Excision of the vocal cords and ventricle was next tried, however, with poor results. Extirpation of the entire arytenoid cartilage was then attempted, but here the animals died of mechanical pneumonia (Schluck-pneumonie) caused by the entrance of saliva and food-particles in respiratory organs. Partial arytenoid extirpation was also tested ; in some cases it gave relief, while in others the respiratory abnormalities were increased. Guenther's last attempt was to detach the vocal ventricle on the diseased side of the larynx and separate the arytenoid cartilage from its thyroidal attachment. This mode of operation also gave variable results, once beneficial then again the reverse. Guenther ascribed poor healing processes as the cause of failure in the majority of cases operated according to the last method. Now, Gerlach appeared in the field ; he, however, condemned operative measures as a means of relieving roaring after having performed a few operations. Stockfleth also tested Guenther's operations ; condemning them, however, after giving them a short trial.

Several other prominent veterinarians experimented with this operation, but each succeeding trial only tended to obscure laryngotomy more and more, until finally it was mentioned only as a historical fact. Until Moeller, in 1885, instituted his experiments, laryngotomy had received absolutely no further attention.

He first tried section of the vocal cords with results similar to those of Guenther. He now devised the following four methods, one giving way to the other, the fourth proving to be the proper one. As will be noticed, method four really belongs to Guenther, but in Moeller's hands, modified and supported by various therapeutical measures, it first gained recognition and prestige.

Method number one consisted in opening the aryteno-thyroid articulation, entrance to the interior of the larynx being effected by an incision of the cricoid cartilage and the first two tracheal rings. Method number two consisted in cutting the posterior crico-arytenoid muscles on the palsied side of the larynx—the idea was that the ensuing connective tissue-growth would contract, and thereby bring the cartilages into their normal position. In method number three the arytenoid cartilage was ligated to the thyroid cartilage by means of sutures. The operation was performed without opening the larynx or trachea. As aforementioned, the first three procedures proved ineffectual. Method number four, which will now be described, is the only one which, in the hands of Moeller, has given satisfactory results. It consists in totally extirpating the arytenoid cartilage. Guenther, who had tested this method, found that the entrance of blood into the respiratory tract during the operation was a great obstacle. This barrier was overcome by Moeller by using the tampon canula (Fig. A). This instrument consists of a long tracheotomy tube, the outer shaft of which is surrounded by a dilatable rubber bulb. The rubber bulb, attached to the tube, is introduced into the trachea and inflated by means of a small air-pump apparatus, which is connected with the bulb by means of a piece of rubber tubing. The bulb should be inflated, the rubber tube tied, to prevent the egress of air, and separated from the air-pump. Through this means, blood-clots, etc., are prevented from entering the lungs. To perform the operation, the animal is to be cast and then brought thoroughly under the action of chloroform,

FIG. A.

Tampon Canula. (Moeller.)

which is most safely and efficiently done by placing *a thin piece of flannel over either one of the nasal openings* and allowing the chloroform to fall in separate and distinct drops, from a drop-bottle, at intervals of one or two seconds upon the flannel. The narcosis being complete, the animal is laid upon its back and kept in position by means of straw proppings. The hair having been shaved and the skin thoroughly cleansed in the region of the larynx, the animal is in readiness for the operation.

Firstly, the skin should be incised, then the sterno-hyoid and sterno-thyroid muscles carefully separated, the third, second and first tracheal rings and the cricoid cartilage incised, the tampoon canula rapidly inserted and the rubber bulb inflated. Care should be taken not to inflate the rubber bulb more than necessary, lest necrosis of the mucous membrane would result. The canula is held in place during the operation by attaching a piece of tape to it, which is held, by an assistant, in a line parallel with the neck. By means of two strong hooks the incised structures are now forced widely apart and the operation can proceed. The arytenoid cartilages should be noticed whether or not they act harmoniously. During even ordinary respiratory movements the palsiedside is easily recognized. However, to further confirm the diagnosis, the larynx should be irritated with some blunt instrument, whereupon a deglutition movement ensues which brings to light any motor deficiency of the laryngeal apparatus which may exist. The extirpation of the arytenoid cartilage is performed with a slightly convexed blunt-pointed bistoury (Fig. B). The blunt end of the bistoury serves also as a probe in separating the cartilage from its muscular attachments. The arytenoid cartilage is extirpated by incision

FIG. B.

FIG. C.

Larynx of horse after separation of the cricoid cartilage and the first three tracheal rings. *t.c.* thyroid cartilage, *v.c.* vocal cord, *l.o.i.* line of incision, *c.c.* cricoid cartilage, *a.c.* arytenoid cartilage, *t.r.* first, second and third tracheal rings. (Moeller.)

commencing at the transverse arytenoid cartilage, and carried as is indicated by the dotted lines in Fig. C. By means of the blunt-pointed bistoury the musculi crico-arytenoidei are detached from the arytenoid cartilage. With a curved scissors the vocal cord is separated from the arytenoid, while the latter is freed from the mucous membrane of the thyro-epiglottidean fold. To effect this, Moeller recommends that the finger of the left hand be introduced into the cul-de-sac of the fold, and by gentle pressure liberating the cartilage from it.

The cartilage should be separated from the thyro-arytenoid muscle, freed from any adherent connective tissue, grasped with a tenaculum and severed completely from the laryngeal wall. Care should be taken that the remaining portion of the arytenoid cartilage does not protrude, lest it form a nucleus for extensive granulations and connective tissue growth.

Prof. Moeller informs me that he has found that suturing of the lacerated mucous membrane greatly facilitates the healing of the parts. After the operation the wound should be thoroughly cleansed with water, and then treated with a 10 per cent. chloride of zinc solution. Following the above procedure, a mass of oakum or jute, consisting of a number of small and delicately rolled balls, sprinkled with tannin and iodoform, should be introduced into the laryngeal cavity. To insure safety and facilitate the removal of the oakum ball, a thin cord should be passed through and around it, and the ends tied around the animal's neck. The skin should be sutured in three places. The tampon canula is held in position by means of two strands of cord or tape, which should be quite firmly tied, displacement being prevented by interlacing them with the mane. The animal should be placed, unhaltered, in a box-stall without bedding, and food is to be withheld for twenty-four hours. After elapse of the *fast* the sutures are to be removed, the oakum extracted, and the wound cavity thoroughly cleansed.

The canula should be removed and replaced by a clean one. After having dilated the rubber bulb of the canula as before described, the animal should be allowed to drink some water from a pail placed upon the floor. The animal's thirst having been quenched, the wound should be irrigated with a 1–1000 sublimate solution, and finally dusted with equal parts of tannin and iodoform. Hay should constitute the only food for three or four days following the operation. After this period the canula can be withheld and soft food, as ground oats and bran, can be fed.

Under daily repeated cleansing the wound should be allowed to heal, which is generally accomplished in from three to four weeks. In eight weeks, or even less, after the operation, the animal can be exercised, beginning with walking exercise and gradually increasing the rate and quantity of work until the animal has developed its full working capacities again. In some cases a slight abnormal sound exists, which, however, generally disappears within five to six months. Some of Prof. Moeller's animals were again at work four weeks after the operation. Of 30 of the first animals operated upon, according to the Moeller method, 22 were entirely *cured*. Five of the remaining eight horses were only partially benefited; two died of septicæmia, while the remaining animal met with an accident, which necessitated its destruction. Since the above 30 operations, 70 additional ones have been performed with but two unfortunate terminations; one animal remaining a roarer, while the other was affected with abnormalities of deglutition.[1]

[1] In the above we have resorted to Moeller's work on "Kehlkopfpfeifen" for the history and the description of the operation.

## Figures and Tables

**Fig. A. f1:**
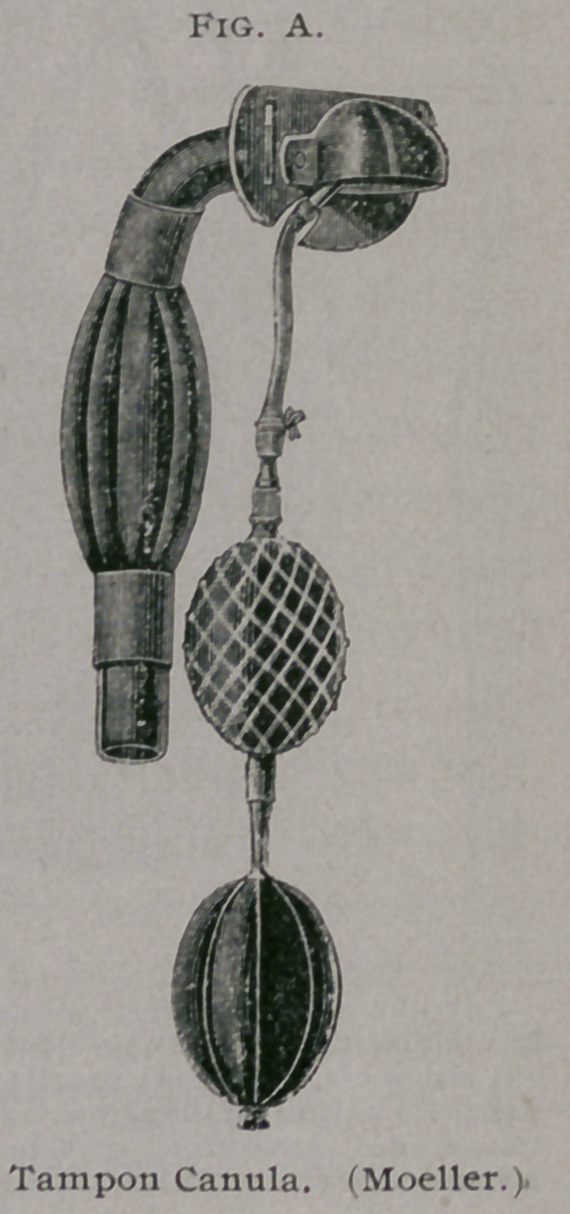


**Fig. B. Fig. C. f2:**